# Study of the Behavior of Square Concrete-Filled CFRP Steel Tubular under a Bending-Torsion Load

**DOI:** 10.3390/polym14071472

**Published:** 2022-04-05

**Authors:** Qing-Li Wang, Hang-Cheng Gao, Kuan Peng

**Affiliations:** 1 School of Civil Engineering, University of Science and Technology Liaoning, Anshan 114051, China; 320183700100@ustl.edu.cn (Q.-L.W.); ghc1112021@163.com (H.-C.G.); 2 School of Mechatronic Engineering, Southwest Petroleum University, Chengdu 610500, China

**Keywords:** CFRP, square concrete-filled steel tube, bending-torsion performance, experimental study, finite element analysis

## Abstract

To study the behavior of square concrete-filled CFRP (carbon fiber polymer) steel tubular under bending-torsional load, nine square section concrete-filled CFRP steel tubular specimens are designed. The T-θ curve and failure mode of square concrete-filled CFRP steel tubular are studied under a bending-torsional load. Based on the test results, a finite element modeling method is proposed by using the finite element software ABAQUS, and the simulation results are compared with the experimental results. The results show that the simulation is in good agreement with the experimental results. On the basis of verifying the reliability of the model, the whole stress process and parameter analysis of the component are studied, and the calculation expression of bearing capacity of square concrete-filled CFRP steel tubular under bending-torsion load is proposed. The predicted specimen-bearing capacity of the proposed calculation expression of the bearing capacity of square concrete-filled CFRP steel tubular under bending-torsion load is basically consistent with the test results.

## 1. Introduction

The steel–concrete composite structure is one of the most widely used composite materials in the world. It is composed of steel and concrete, which takes advantage of the compressive properties of concrete and the tensile properties of steel [1,2,3]. That is, the tensile capacity of concrete is poor, but the use of steel just makes up for this disadvantage. The two materials complement each other, so as to utilize their respective advantages to the greatest extent. This composite structure is not only convenient for construction, but also achieves the objectives of compressing cost, reducing component weight and shortening the construction period. Therefore, steel–concrete composite structures can be widely used in practical engineering [4,5,6]. Carbon fiber reinforced polymer (CFRP), one of the most widely used fiber materials, is characterized as light-weight and high-strength. In construction engineering, CFRP, one of the current research hotspots, can be wrapped on the outside of the concrete-filled steel tube to restrain the concrete together with the steel tube, delay the buckling of the member and strengthen and repair the damaged member [7,8].

Al Mekhlafi et al. [9] carried out an eccentric compression test of CFRP confined concrete-filled stainless steel tubular short columns, with a total of 12 short columns. The test parameters included the thickness and eccentricity to outer diameter ratio (E/D) of CFRP, and established a three-dimensional finite element model for a numerical simulation on the basis of the test. The results show that the restraint of CFRP can effectively improve the ultimate eccentric compressive strength of CFRP concrete-filled steel tubular short columns. When CFRP breaks, the member begins to fail and an axial force moment interaction model is proposed. Tao et al. [10] used CFRP to repair the concrete-filled steel tube affected by fire, and then carried out the test of axial compression performance and bending resistance. The test designed two cases: no reinforcement in case of fire, and reinforcement in case of fire. The results show that the bearing capacity and stiffness of the fire-resistant concrete-filled steel tubular repaired with CFRP was improved to a certain extent, but the repair effect of the beam is not obvious compared with the short column. Liu et al. [11] designed a total of 11 FRP (fiber polymer) concrete-filled steel tubular short columns when studying the axial compression bearing capacity of FRP concrete-filled steel tubular. The type and quantity of FRP, the thickness of the steel tube and the strength grade of concrete are the main variation parameters of the specimens in the test. The experimental results show that the bearing capacity of concrete-filled steel tubular wrapped with FRP is higher than that of concrete-filled steel tubular, which shows that the use of FRP is meaningful. Guan et al. [12] carried out a numerical simulation of CFRP concrete-filled steel tubular columns under an impact load. The three-dimensional finite element model is established and verified by the test data, and the parameter analysis is carried out to reveal the influence of geometric and material parameters on the impact resistance of CFRP concrete-filled steel tubular columns. Wang et al. [13] carried out numerical simulation research on CFRP concrete-filled steel tubular columns under a combined compression bending torsion load. The three-dimensional finite element model is established and verified by the test data. Based on the finite element simulation, the typical failure mode and the load deformation relationship of the components are analyzed.

At present, the research on concrete-filled CFRP steel tubular mostly focuses on single load. Based on this, the flexural-torsional load test of concrete-filled CFRP steel tubular is designed to study the flexural-torsional properties of square section concrete-filled CFRP steel tubular. Based on the test results, the corresponding model is established using ABAQUS finite element analysis software, and the established finite element model is used to study the influence of steel yield strength, steel ratio, number of CFRP layers, concrete compressive strength and moment ratio on the bearing capacity of the member, and to analyze the whole process of the stress on each component material of the member. Finally, based on the test and finite element method, the functional expression of the bearing capacity of concrete-filled CFRP steel tubular under bending and torsional load is derived.

## 2. Design and Material Properties of Specimen

### 2.1. Design of Specimen

The flexural torsional static performance tests of nine square concrete-filled CFRP steel tubular specimens were prepared. The longitudinal CFRP layers (*m*_l_), transverse CFRP layers (*m*_t_) and bending moment ratio (*m*) were selected as the main parameters. The length *L* of all specimens was 540 mm, the outer length *B*_s_ of steel tubes was 120 mm, and the wall thickness *t*_s_ of steel tubes was 3 mm. The specific parameters are shown in Table 1. Firstly, concrete-filled steel tube was prepared according to Han LH [8]. After that, acetone was used to clean the welding slag and oil on the steel tube surface. The viscose was evenly applied to the surface of the steel tube, and part of the viscose was evenly applied to the surface of CFRP. The bubbles were removed by scraping to ensure that the adhesive completely penetrated into CFRP. The sequence of pasting CFRP is as follows: first, paste the longitudinal CFRP. When its surface is dry, then paste the transverse CFRP, and check that the lap length of the transverse CFRP is 150 mm. Finally, apply a layer of adhesive to the outer layer to make it completely cured within one week.

### 2.2. Material Properties

The yield strength (*f*_y_), tensile strength (*f*_u_), elastic modulus (*E*_s_), Poisson’s ratio (*v*_s_) and elongation of steel (*ε*′) used in specimens are shown in Table 2. The performance indexes of concrete used in concrete-filled CFRP steel tubular bending torsion specimen and CFRP are shown in Table 3 and Table 4. CFRP performance test method according to Wang [14]. All specimens use a JGN-C building structure adhesive, produced by the Liaoning Academy of construction sciences, Shenyang, Liaoning, China, as the special dipping adhesive for pasting carbon fiber cloth, and its properties can be seen in Table 5.

**Figure 1:**
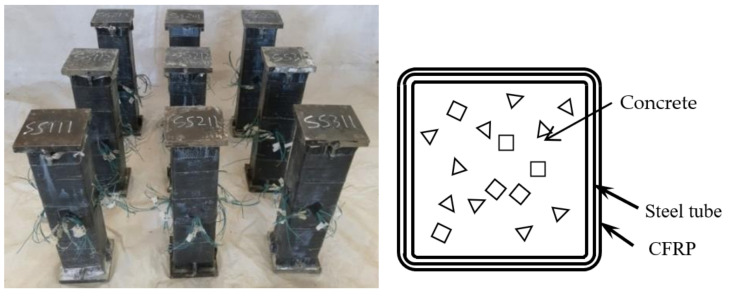
Diagram of S-CF-CFRP-ST and members before test.

The section diagram of square concrete-filled CFRP steel tubular and members before the flexural torsional test are shown in Figure 1.

### 2.3. Loading Test and Measurement

**Figure 2:**
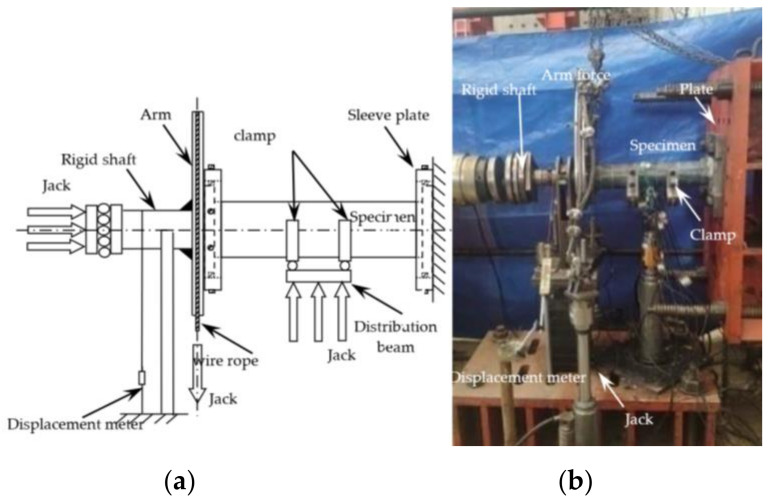
Diagrammatic sketch and physical figure of loading equipment. (**a**) Diagrammatic sketch of loading equipment. (**b**) Physical figure of loading equipment.

The test was completed in the Structural Engineering Laboratory of Shenyang Architecture University. A jack, a load distribution beam and a clamp were added to the specimen to apply the bending moment. A concentrated force with equal values and the same direction is applied to one-third of both sides of the specimen through the distribution beam to achieve the effect of applying the bending moment. The loading equipment and its schematic diagram are shown in Figure 2.

In order to ensure that the specimen can be stressed uniformly, preload within the elastic range is carried out, and the preload value is 30% of the estimated bearing capacity. The graded loading system is adopted in the test. Within the elastic range, each level of loading is 1/10 of the estimated ultimate bearing capacity, and the next level of loading is carried out after holding the load for 2~3 min. When the tension reaches about 70% of the estimated ultimate bearing capacity, the loading of each stage is reduced to 1/15~1/20 of the estimated ultimate bearing capacity. After yielding, the test piece shall be loaded slowly and continuously until the jack reaches the maximum range, and the test shall be stopped.

### 2.4. Failure Mode of Materials

**Figure 3:**
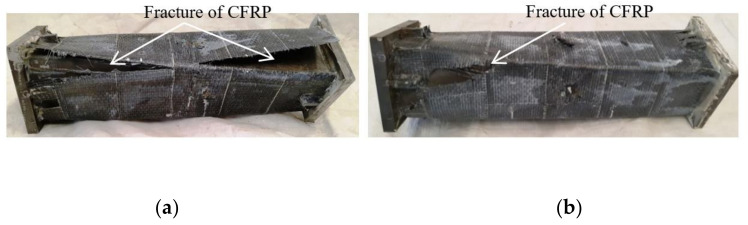
Failure model of CFRP. (**a**) SBT111 specimen. (**b**) SBT211 specimen.

**Figure 4:**
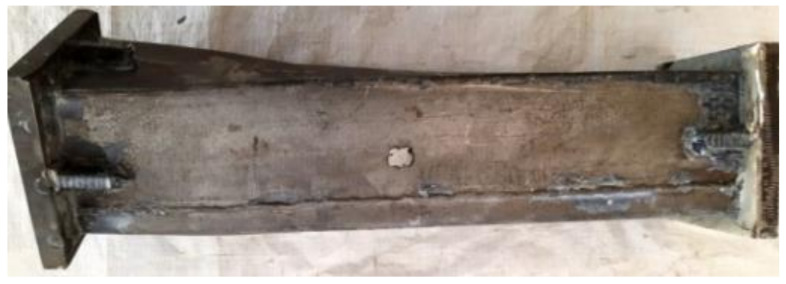
Failure model of steel tube.

**Figure 5:**
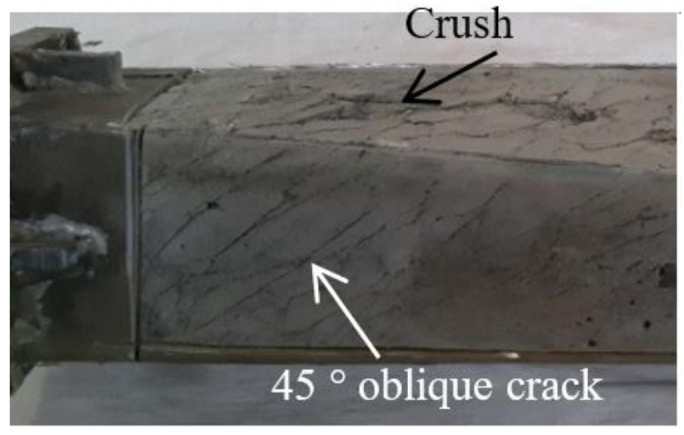
Failure model of concrete.

**Figure 6:**
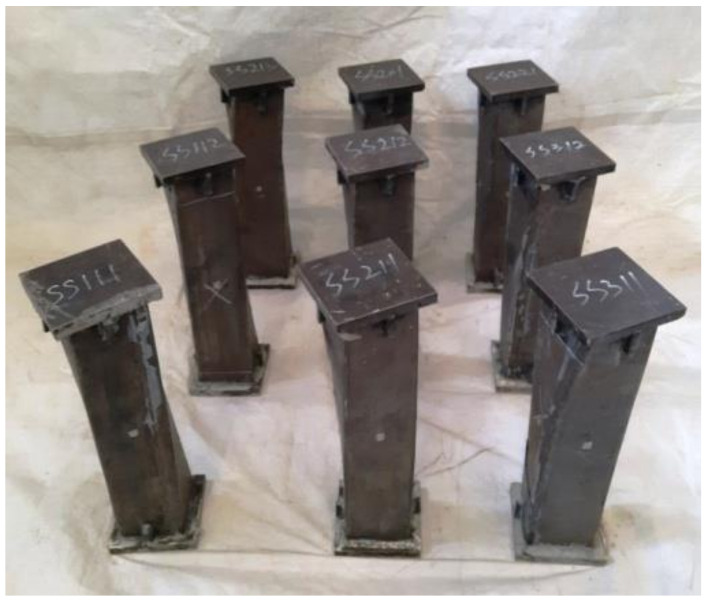
Square section specimen after flexural-torsional test.

The CFRP failure mode, steel tube failure mode and concrete failure mode of the square section specimen are shown in Figure 3, Figure 4 and Figure 5, respectively. A transverse and longitudinal CFRP fracture can be seen in the bending area of the specimen with a small moment ratio; the fracture develops from the end of the specimen to the middle of the specimen. For the specimens with a large moment ratio, the cracking degree of CFRP is light because the bending moment is large and has a certain restraint effect on the members. It can be seen that the steel tube is not cracked, and the specimen has obvious torsional deformation after loading. A large number of 45° inclined cracks appear in the concrete, and some concrete in the flat area is crushed, which indicates that the steel tube and concrete are mainly subjected to torsional damage. Figure 6 shows all specimens after the flexural-torsional test.

### 2.5. Test Results and Preliminary Analysis

#### T-θ Curves

**Figure 7:**
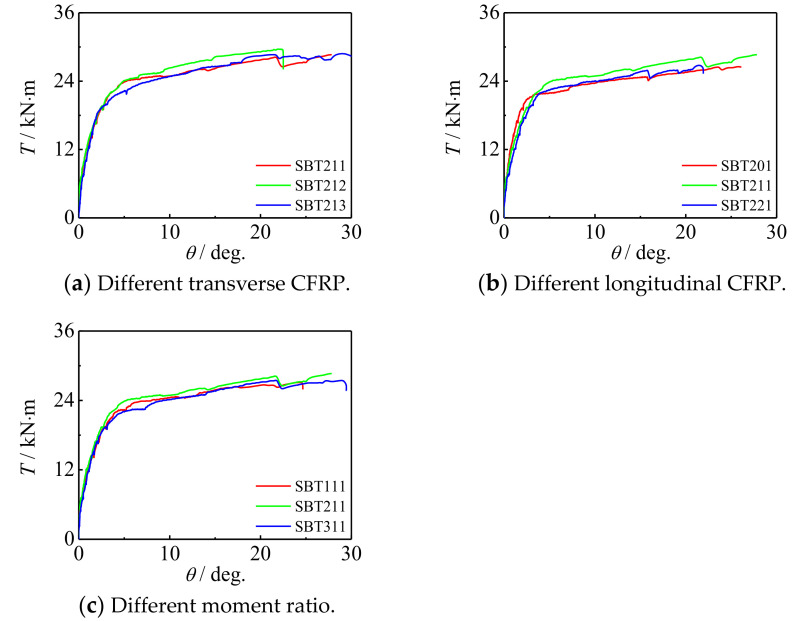
*T*-*θ* curves of square section specimen.

Figure 7 shows the *T*-*θ* curve of a square concrete-filled CFRP steel tubular flexural torsional performance specimen. It can be seen that the curve develops linearly at the initial stage of loading, and the specimen is in the elastic stage. Then, the curve enters the elastic-plastic stage. The CFRP of the square specimen is broken in a large area and failed. The curve rises gradually after the sudden drop, indicating that the component can still maintain good mechanical performance after the CFRP is fractured. In the later stage of loading, it can still maintain a certain torque after a large angle, which indicates that CFRP has a certain restraint effect on the specimen, and the member shows good ductility.

## 3. Finite Element Simulation

### 3.1. Finite Element Model

**Figure 8:**
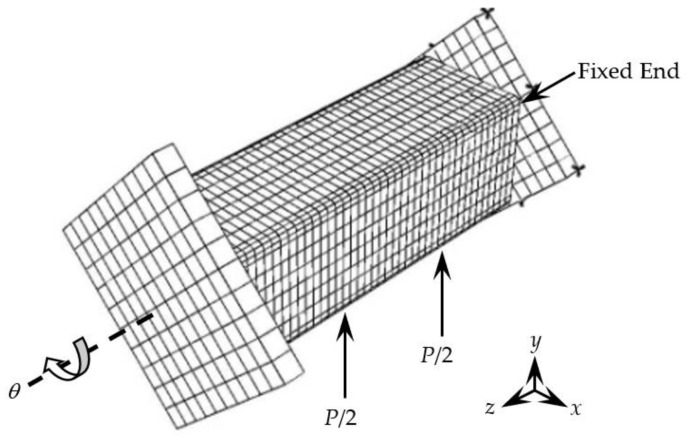
The boundary conditions of finite element simulation of specimen.

The stress–strain relationship of the steel tube and concrete used in the finite element simulation of concrete-filled CFRP steel tube bending torsion member is suggested by Han [15]. The stress–strain relationship of CFRP is suggested by references [14,15,16,17]. When the strain of the transverse CFRP reaches its fracture strain *ε*_cftr_ (3000 µε) or the strain of longitudinal CFRP reaches its fracture strain *ε*_cflr_ (3000 µε), the transverse restraint effect or longitudinal reinforcement effect on the steel pipe will be lost, respectively. Figure 8 shows the boundary conditions of the finite element simulation of square CFRP concrete-filled steel tubular flexural torsional members. One side is used as the fixed end to restrict the displacement and rotation angle in *x*, *y* and *z* directions. The bending moment is converted into two concentrated forces according to the bending moment ratio and applied to the middle of the lower span of the member. The other side is used as the loading end to apply the angle.

### 3.2. Comparison between Simulation Results and Test Results

#### 3.2.1. *T*-*θ* Curves Comparison

**Figure 9:**
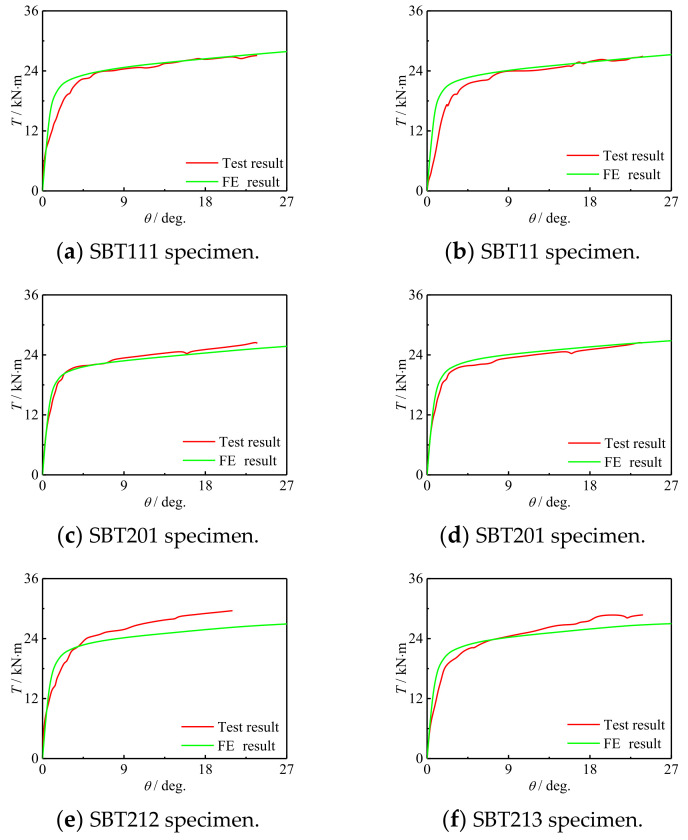

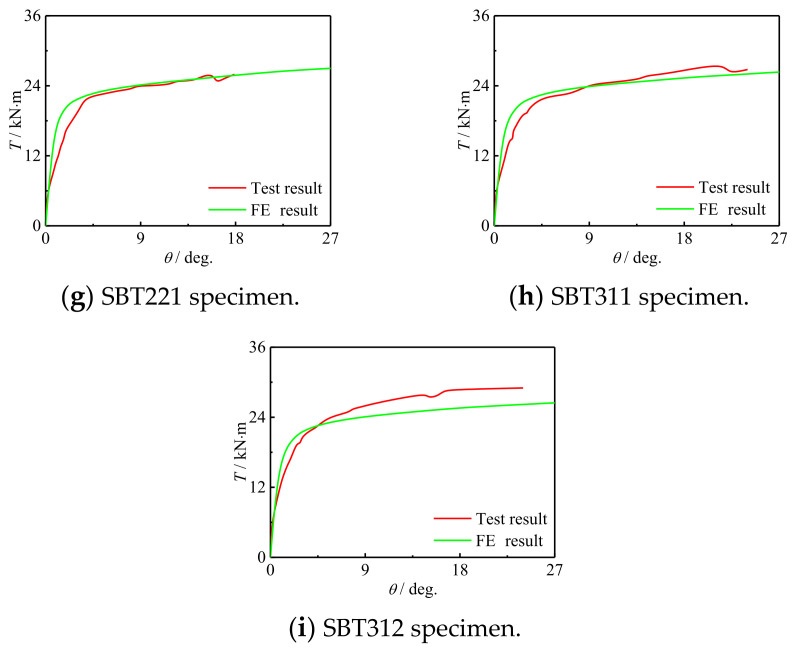
The comparison *T*-*θ* curve.

Figure 9 shows the comparison of the *T*-*θ* curve between the simulation results and the test results. It can be seen that the early stiffness and ultimate bearing capacity of the curve established by the finite element model are in good agreement with the test. It can be inferred that the simulation results are in good agreement with the *T*-*θ* curve of the test results.

#### 3.2.2. Failure Mode Comparison

**Figure 10:**
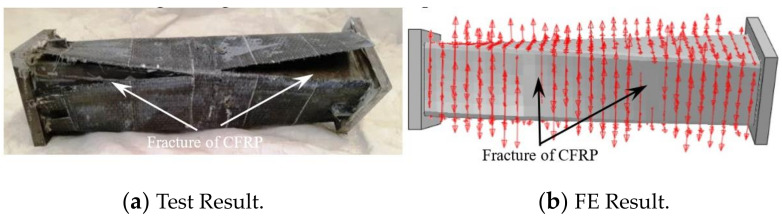
Failure mode of transverse CFRP.

Figure 10 shows the failure mode of the transverse CFRP of the square CFRP concrete-filled steel tubular flexural torsional performance specimen (the arrow in the figure indicates the transverse CFRP that has not been broken). The finite element results show that CFRP cracks from both ends to the middle of the member, and the failure mode is in good agreement with the experimental CFRP fracture simulation results.

**Figure 11:**
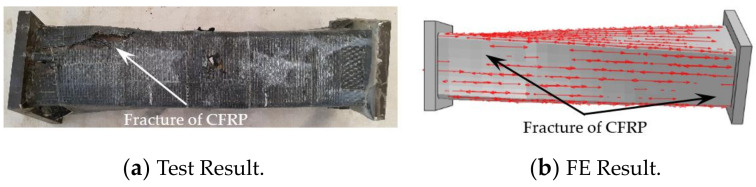
Failure mode of longitudinal CFRP.

Figure 11 shows the failure mode of longitudinal CFRP of a square CFRP concrete-filled steel tubular flexural torsional performance specimen (the arrow in the figure indicates the longitudinal CFRP that has not been broken). As can be seen in Figure 11, the finite element mode shows that the longitudinal CFRP cracks from the upper and lower ends to the middle, respectively, indicating that the finite element results are in good agreement with the test results.

**Figure 12:**
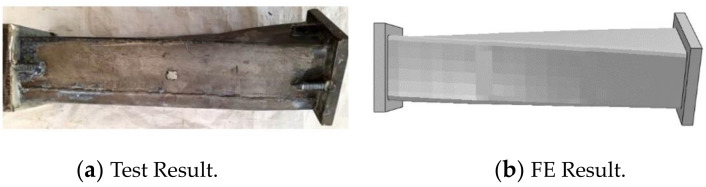
Failure mode of steel tube.

Figure 12 shows the failure mode of a square CFRP concrete-filled steel tubular flexural torsional performance specimen. It can be seen from Figure 12 that the failure mode of steel pipe simulated by finite element is basically consistent with that of the test. From the test and simulation results, it can be seen that the steel pipe is mainly subjected to torsional failure.

**Figure 13:**
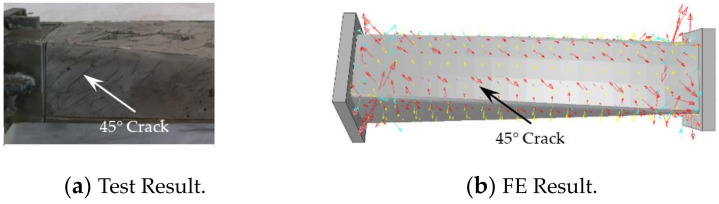
Failure mode of concrete.

Figure 13 shows the failure mode of concrete inside the square CFRP concrete-filled steel tubular flexural torsional performance specimen, in which the arrow in the figure indicates the concrete crack. It can be seen from Figure 13 that concrete and steel pipe are the same, which are mainly subject to torsional deformation and produce 45° cracks, and the failure mode of concrete simulated by finite element is basically consistent with that of the test. Through the *T*-*θ* curve and the failure of each component material, it can be seen that the finite element results are in good agreement with the test results, which shows that the proposed numerical simulation method for the study of flexural and torsional properties of square concrete-filled CFRP steel tubular specimens is reasonable.

## 4. Analysis of the Whole Process of Stress

**Figure 14:**
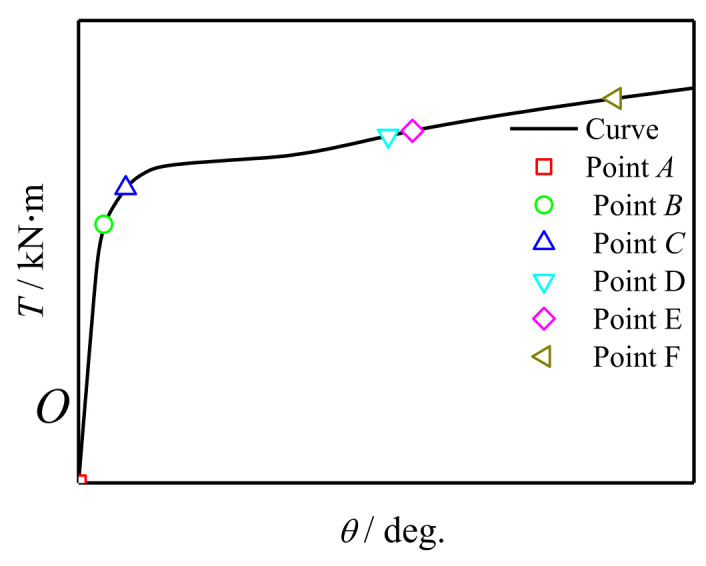
Typical *T-θ* curve of specimen.

Figure 14 shows the typical *T*-*θ* curve of concrete-filled CFRP steel tubular flexural torsional members. A total of six feature points are selected for the curve: point *O* corresponds to the point after the bending moment and before the torque is applied, point *A* corresponds to concrete cracking, point *B* corresponds to the steel reaching yield strength and point *C* corresponds to the transverse CFRP fracture, point *D* corresponds to the longitudinal CFRP fracture. The corresponding component at point *E* reaches a 15° angle.

These six characteristic points are used to analyze its working mechanism in the whole stress process. Calculation parameters: *L* = 540 mm, *B*_s_ = 120 mm, *t*_s_ = 3 mm, *m* = 0.1, *f*_cu_ = 40 MPa (*f*_ck_ = 26 MPa), *f*′_c_ = 35 MPa, *f*_y_ = 345 MPa, *ξ*_s_ = 1.356, *ξ*_cf_ = 0.106, *η* = 0.124.

### 4.1. Stress of Concrete

**Figure 15:**
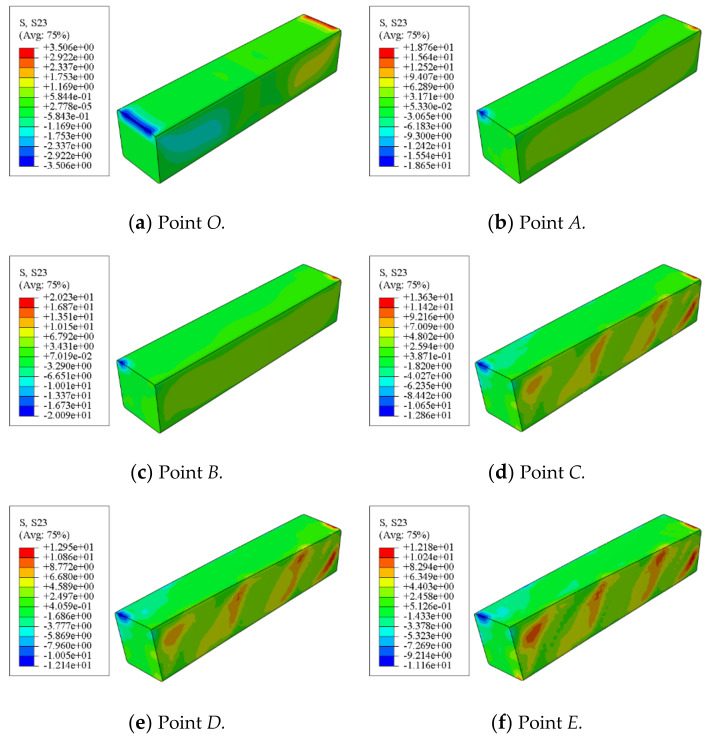
Shear stress distribution of concrete.

Figure 15 shows the shear stress distribution of concrete in a square concrete-filled CFRP steel tubular flexural torsional members. It can be seen that the shear stress of the square members is roughly antisymmetric along the length direction. During the whole loading process, the maximum shear stress is always at the end of the concrete.

**Figure 16:**
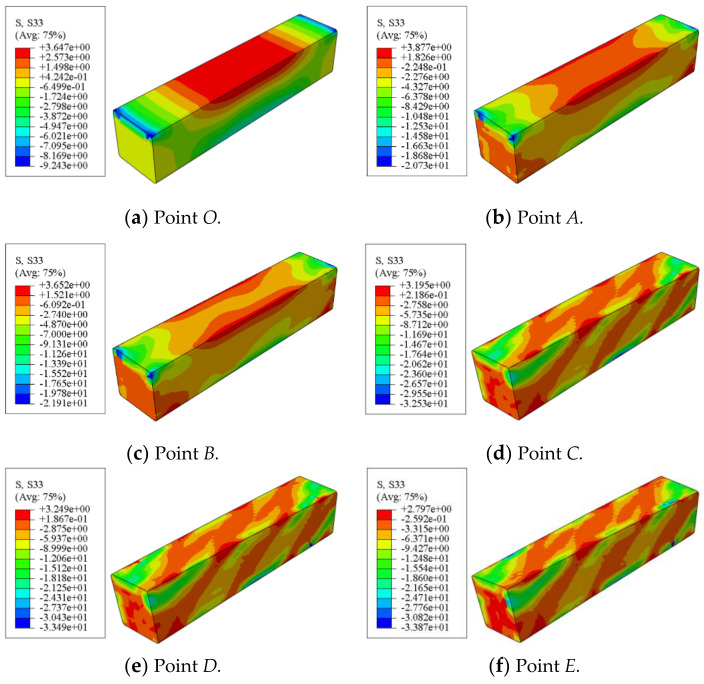
Longitudinal stress distribution of concrete.

Figure 16 shows the longitudinal stress distribution of concrete in a square concrete-filled CFRP steel tubular flexural torsional members. It can be seen that at points *O*~*B*, the longitudinal stress of concrete is symmetrically distributed left and right along the length direction. In the subsequent loading process, the longitudinal stress of the square member is basically distributed in the 45° direction along the length direction, which shows that from the whole process analysis, the concrete inside the member is mainly affected by torsional deformation.

### 4.2. Stress of Steel Tube

**Figure 17:**
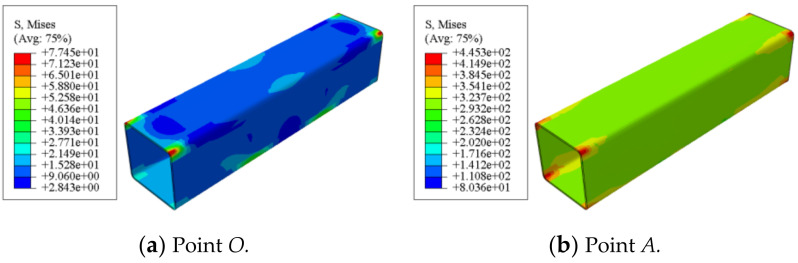

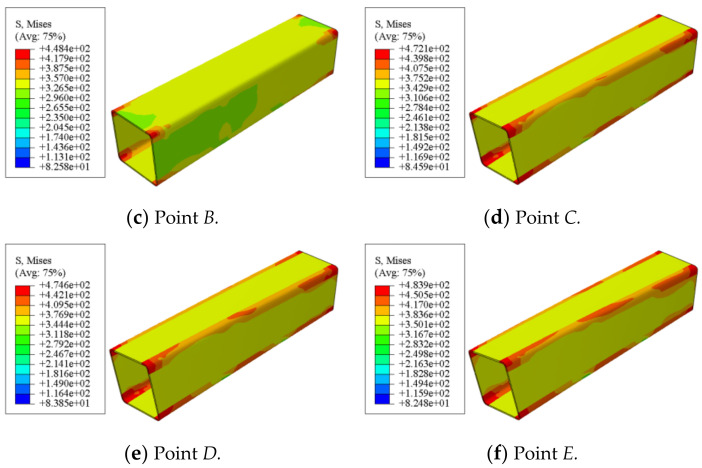
Mises stress distribution of member.

Figure 17 shows the Mises stress distribution of square concrete-filled CFRP steel tubular flexural torsional member. It can be seen that at point *A*, the steel is still in the elastic stage, the stress is still very small, and the stress at the steel plate of the component is smaller than that at the corner. When the steel is loaded to point *C*, the steel is elastic to yield, the stress of the steel pipe increases greatly, the steel plate of the component begins to yield and the maximum stress appears at the corner. After point *C*, the steel pipe gradually strengthened and the stress continued to increase. In the whole loading process, the maximum Mises stress of the square member is always concentrated at the corner due to the stress concentration.

### 4.3. Stress of CFRP

#### Transverse CFRP Stress

**Figure 18:**
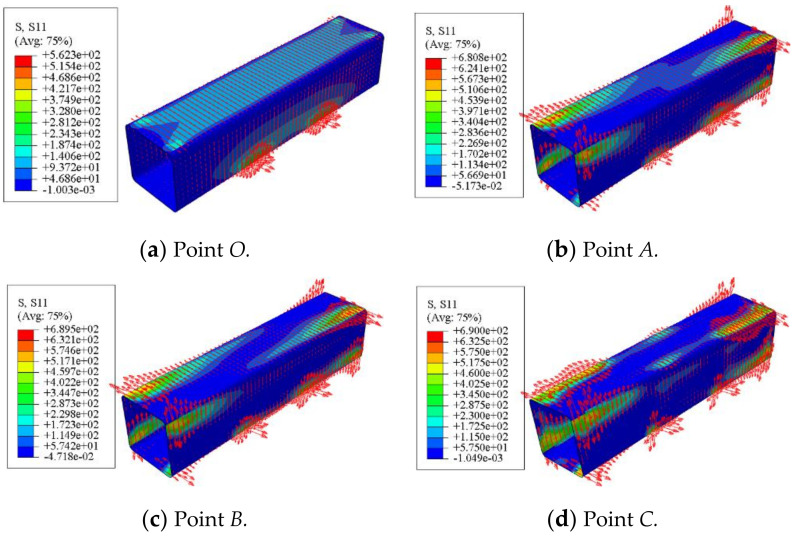

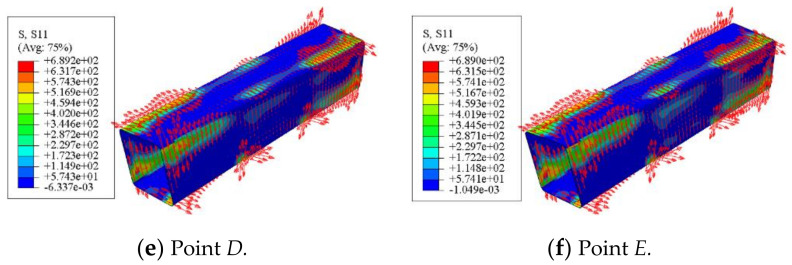
Stress distribution of transverse CFRP.

Figure 18 shows the transverse CFRP stress distribution of square concrete-filled CFRP steel tubular flexural torsional members. It can be seen that at point *O*, the member deformation is very small and the transverse CFRP stress is also very small. With the application of torque, the stress of transverse CFRP increases gradually in the process of loading to point *A* and even point *B.* When loaded to point *C*, the transverse CFRP stress near the corner of the steel pipe reaches 690 MPa and begins to fracture. After that, the transverse CFRP fracture area of the square member increases gradually from the middle section to the end plate and from the corner to the midpoint of the plate, which shows that the transverse CFRP plays a good role in restraining the member.

**Figure 19:**
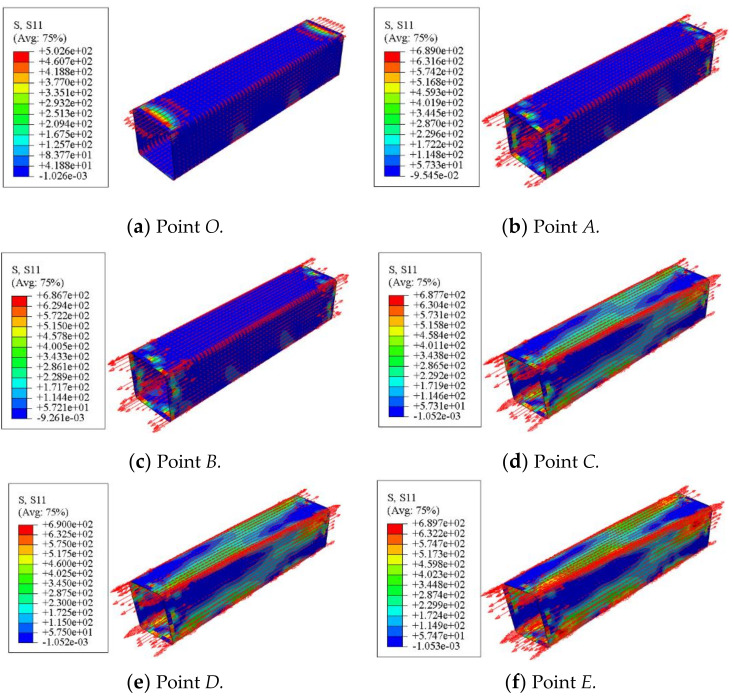
Stress distribution of longitudinal CFRP.

The longitudinal stress distribution of the concrete-filled CFRP steel tubular flexural torsional member is shown in Figure 19. At point *O*, the member deformation is small and the longitudinal CFRP stress is also small. With the progress of loading, the longitudinal CFRP stress increases gradually. When the member is loaded to point *D*, when the longitudinal CFRP stress at the corner reaches 690 MPa, the CFRP begins to fracture and is also mainly concentrated at the corner.

## 5. Parameter Analysis

The possible parameters affecting the *T*-*θ* curve of the concrete-filled CFRP steel tubular bending torsion members include the number of CFRP layers, steel yield strength, concrete strength, bending moment ratio and steel ratio. The influence laws of the above parameters are analyzed through calculation examples (*L* = 540 mm, *B*_s_ = 120 mm, *t*_s_ = 3 mm, *m* = 0.1, *f*_cu_ = 40 MPa, *f*_y_ = 345 MPa, *α* = 0.105, *ξ*_s_ = 1.356, *ξ*_cf_ = 0.106, *η* = 0.124).

### 5.1. Influence of CFRP Layers

**Figure 20:**
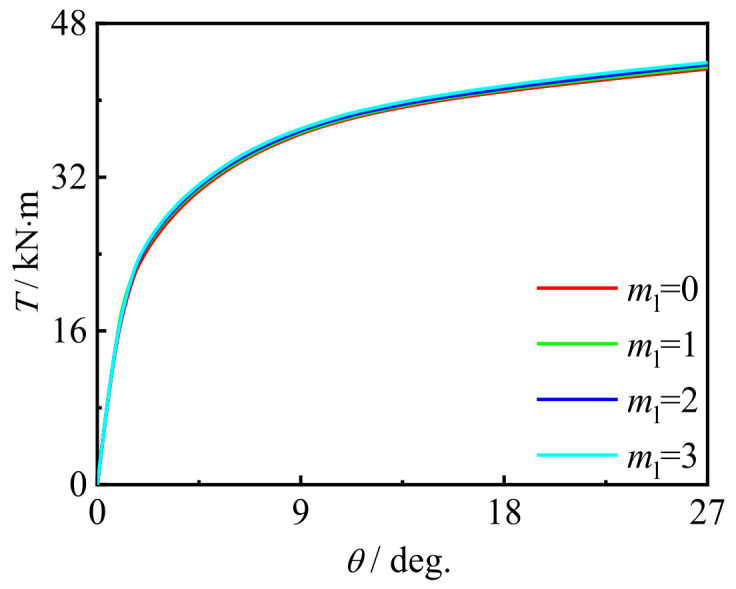
Effect of *m*_l_ on *T*-*θ* curve.

**Figure 21:**
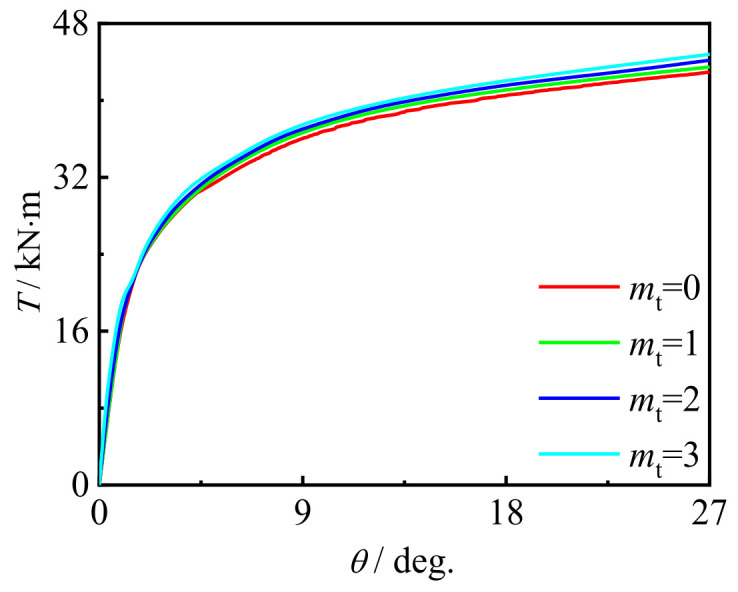
Effect of *m*_t_ on *T*-*θ* curve.

Figure 20 and Figure 21 show the influence of the number of longitudinal CFRP layers and transverse CFRP layers on the *T*-*θ* curve of concrete-filled CFRP steel tubular flexural torsional performance members, respectively. It can be seen that with the increase in the number of CFRP layers, the curve shape and initial stiffness do not change significantly, and the bearing capacity of members increases slightly. This is because with the increase in the number of CFRP layers, the ability of members to be restrained is improved, and the macroscopic performance is that the bearing capacity of members increases, which is also consistent with the test results.

### 5.2. Effect of Material Strength

**Figure 22:**
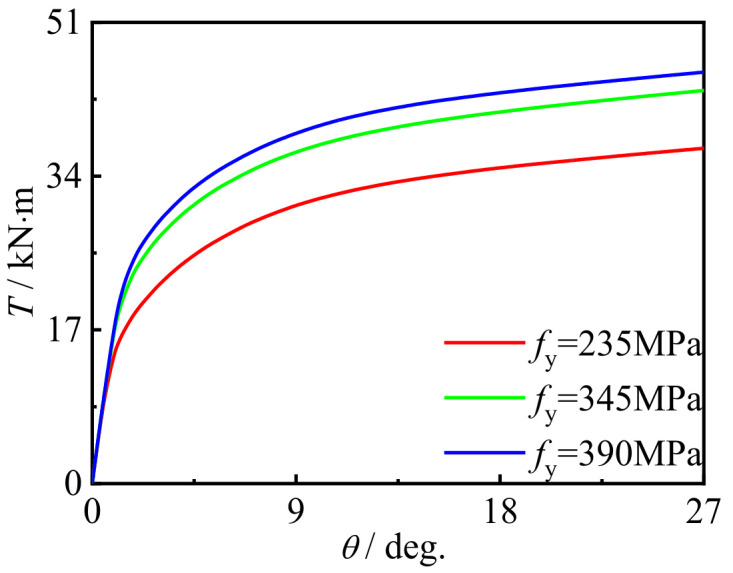
Effect of *f*_y_ on *T*-*θ* curve.

**Figure 23:**
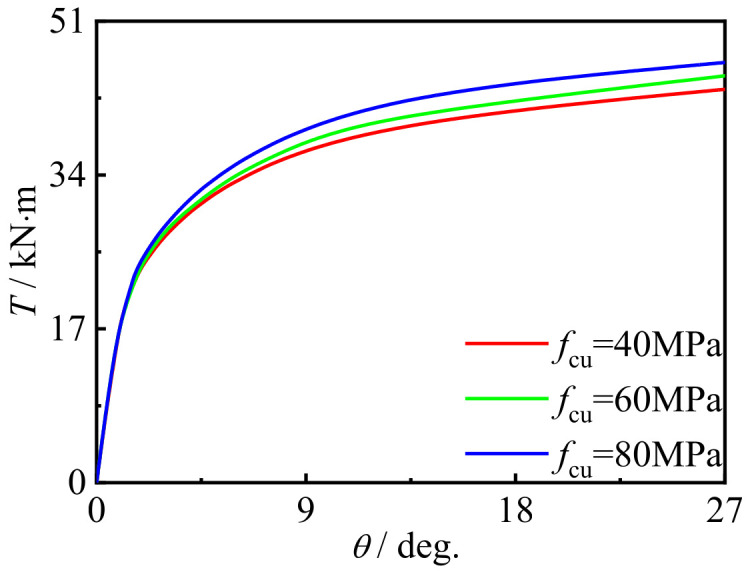
Effect of *f*_cu_ on *T*-*θ* curve.

Figure 22 and Figure 23 show the effects of steel yield strength and concrete strength on the *T*-*θ* curve of concrete-filled CFRP steel tubular flexural torsional performance members, respectively. It can be seen that with the improvement of material strength, there is no obvious change in curve shape and initial stiffness, and the bearing capacity of members is significantly improved. This is because the steel strength and concrete strength are important factors determining the overall strength of the member. Therefore, the strength improvement of these constituent materials will significantly improve the strength of concrete-filled CFRP steel tubular flexural torsional members.

### 5.3. Influence of Bending Moment Ratio

**Figure 24:**
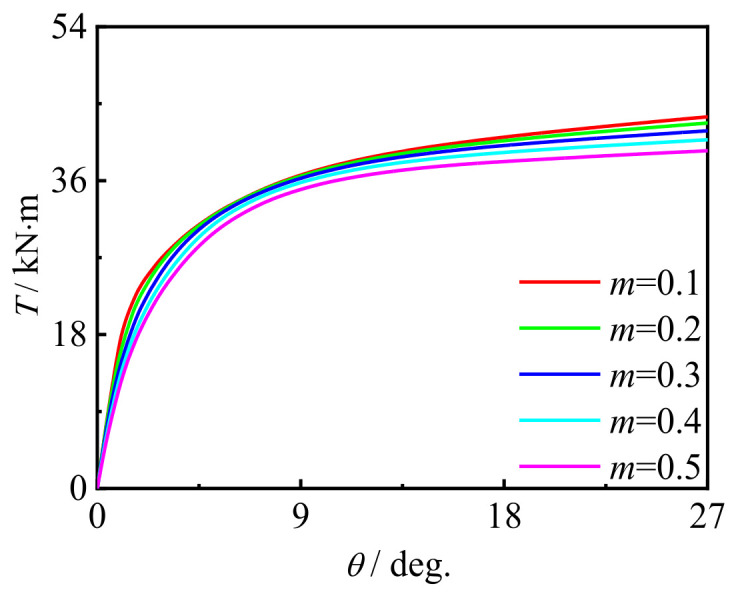
Effect of *m* on *T*-*θ* curve.

Figure 24 shows the effect of the moment ratio on *T*-*θ* curve of concrete-filled CFRP steel tubular flexural torsional member. It can be seen that with the increase in the moment ratio, the curve shape and initial stiffness do not change significantly, and the bearing capacity of members decreases slightly. This is because with the increase in the moment ratio, the damage increases, resulting in the reduction in the bearing capacity of the member.

### 5.4. Effect of Steel Ratio

**Figure 25:**
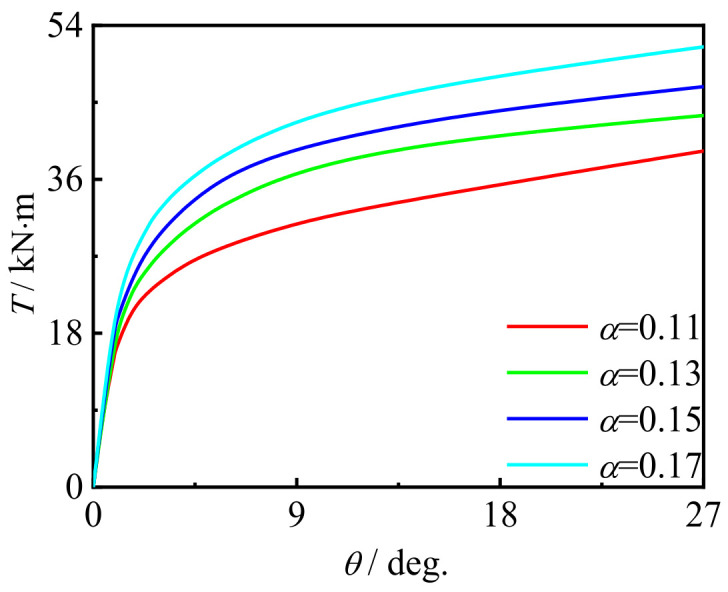
Effect of *α* on *T*-*θ* curve.

Figure 25 shows the effect of steel ratio on the *T*-*θ* curve of concrete-filled CFRP steel tubular flexural torsional member. It can be seen that with the increase in the steel ratio, the early stiffness and member bearing capacity are significantly improved. This is because, with the increase in the steel ratio, the brittleness and strength of steel are significantly improved, which leads to the increase in the early stiffness and maximum bearing capacity of members.

## 6. Bending-Torsion Correlation Equation of Bearing Capacity

### 6.1. Bending-Torsion Correlation Equation

**Figure 26:**
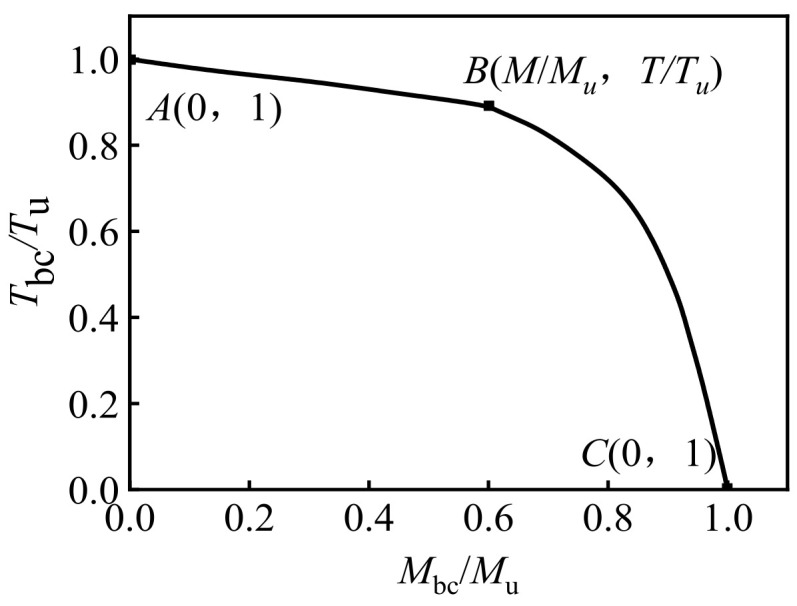
Typical *M*/*M*_u_-*T*/*T*_u_ curve of concrete-filled CFRP steel tubular flexural torsional members.

Figure 26 shows the typical *M*/*M*_u_-*T*/*T*_u_ curve of concrete-filled CFRP steel tubular flexural torsional members.

Using a large number of calculations on the *T-θ* curve of concrete-filled CFRP steel tubular bending torsion members, the calculation parameters (scope of application) are obtained: *f*_y_ = 235 MPa~390 MPa, *f*_cu_ = 30 MPa~80 MPa, *α* = 0.07~0.21, *ξ*_s_ = 0.8~2.5, ξ_cf_ = 0~1.3, *η* = 0~0.7. It is defined that the torque corresponding to the strain of the outermost fiber of the steel tube in the tensile area of CFRP concrete-filled steel tubular flexural torsional member reaches *ε*_max_ is the torsional bearing capacity, in which the value of *ε*_max_ is:

*ε*_max_ = 2837 + 166,800/D_s_ (1)

The *M*/*M*_u_-*T*/*T*_u_ curve can be roughly divided into two parts, and its expression is as follows:

(1) Stage of *A*-*B* (0.1 ≤ *M*/*M*_u_ < 0.7)

(2) $$\frac{T}{T_{u}}+\frac{aM}{M_{u}}=1$$

(2) Stage of *B*-*C* (0.7 ≤ *M*/*M*_u_ < 0.9)

(3) $$-{\left(\frac{M}{M_{u}}\right)}^{2}+\frac{bM}{M_{u}}+\frac{T}{T_{u}}=1$$

where *a* = 0.1326, *b* = 0.9781.

### 6.2. Validation of Expressions

**Figure 27:**
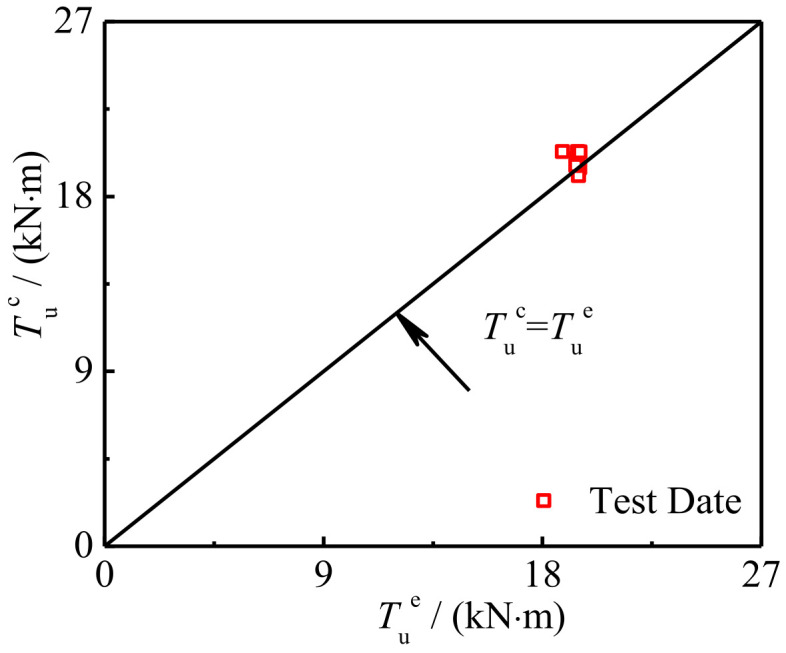
Comparison between *T*_u_^c^ and *T*_u_^e^ of specimen.

Figure 27 shows the comparison between the calculation results *T*_u_^c^ of torsional bearing capacity and the test and finite element results *T*_u_^e^. The average value of *T*_u_^c^/*T*_u_^e^ of components is 1.025 and the mean square deviation is 0.029. It can be seen that the calculated results are in good agreement with the experimental results.

## 7. Conclusions

(1) The *T*-*θ* curve of a concrete-filled CFRP steel tube can be divided into elastic stage, elastic–plastic stage and plastic stage. The specimen can still maintain a certain bearing capacity after large deformation, indicating that the failure of the member belongs to ductile failure. The steel tube and CFRP can work together, and the specimen CFRP breaks in a large area after loading;
(2) The *T*-*θ* curve and failure mode of the specimen are simulated by ABAQUS, and the simulation results are in good agreement with the experimental results. The shear stress of concrete in the member is roughly antisymmetric along the length direction. In the whole loading process, the maximum Mises stress of the steel pipe is always concentrated at the corner. The stress of transverse CFRP and longitudinal CFRP increases gradually with the increase in the load and fails when the deformation increases to a certain extent. Transverse CFRP has a good restraining effect on the specimen;
(3) The parameter analysis results show that the increase in the CFRP layers, material strength, bending moment ratio and steel ratio does not change the shape and initial stiffness of CFRP concrete-filled steel tubular *T*-*θ* curve. With the increase in concrete strength, steel yield strength and steel ratio, the member bearing capacity increases significantly, increases slightly with the increase in the CFRP layers, and decreases slightly with the increase in the moment ratio.
(4) A correlation equation for the bearing capacity of CFRP concrete-filled steel tubular flexural torsional members is proposed. The bearing capacity calculated by this correlation equation is in good agreement with the experimental results.

## Figures and Tables

**Table 1:** Parameters of S-CF-CFRP- ST specimens.

No.	Number	*m*	*M*_l_/Layers	*m*_t_/Layers	*ξ*_cf_	*ξ*	*η*_cf_
1	SFT111	0.1	1	1	0.106	1.253	0.092
2	SFT211	0.2	1	1	0.106	1.253	0.092
3	SFT311	0.3	1	1	0.106	1.253	0.092
4	SFT112	0.1	1	2	0.212	1.359	0.092
5	SFT212	0.2	1	2	0.212	1.359	0.092
6	SFT312	0.3	1	2	0.212	1.359	0.092
7	SFT213	0.2	1	3	0.317	1.464	0.092
8	SFT201	0.2	0	1	0.106	1.253	0
9	SFT221	0.2	2	1	0.106	1.253	0.184

**Table 2:** Material properties of steel.

*f*_y_/MPa	*f*_u_/MPa	*E*_s_/GPa	*v*_s_	*ε*′/%
291.78	456.49	210.16	0.31	27.7

**Table 3:** Material properties of concrete.

*E*_c_/MPa	*f*_cu_/MPa	*f*′_c_/MPa
36000	62.5	49.38

**Table 4:** Material properties of CFRP.

Thickness (mm)	*E*_cf_ (GPa)	Transverse CFRP Fracture Strain *e*_cftr_ (me)	Longitudinal CFRP Fracture Strain *e*_cflr_ (me)
0.111	230	3000	3000

**Table 5:** Properties of JGN-C building structure adhesive.

Technical Performance Index	Measurement Result
Metal tensile strength/MPa	>20.0
Metal bond tensile strength/MPa	≥30.0
Bond tensile strength of concrete/MPa	≥2.5
Tensile modulus of elasticity/MPa	≥15,000
elongation/%	≥1.5

## Data Availability

Data is contained within the article or supplementary material.

## References

[1] Li X., Hu Z., Zhou J. (2020). Calculation method of axial compressive capacity of circular arc scfst short columns restrained by CFRP. Struct. Engineer..

[2] Jiao C.J., Li S.C. (2021). Axial compression behavior of CFRP confined steel tube reactive powder concrete short columns. J. Compos. Mater..

[3] Liang J.F., Lin S.Q., Li W., Liu D.W. (2021). Axial compressive behavior of recycled aggregate concrete-filled square steel tube stub columns strengthened by CFRP. Structures.

[4] Tang H.Y., Chen J.L., Fan L.Y., Sun X.J., Peng C.M. (2020). Experimental investigation of FRP-confined concrete-filled stainless steel tube stub columns under axial compression. Thin-Walled Struct..

[5] Zhang Y., Wei Y., Zhao K., Ding M., Wang L. (2020). Analytical model of concrete-filled FRP-steel composite tube columns under cyclic axial compression. Soil Dyn. Earthq. Eng..

[6] Martinelli E., Hosseini A., Ghafoori E., Motavalli M. (2019). Behavior of prestressed CFRP plates bonded to steel substrate: Numerical modeling and experimental validation. Compos. Struct..

[7] Jin L., Fan L., Li P., Du X. (2019). Size effect of axial-loaded concrete-filled steel tubular columns with different confinement coefficients. Eng. Struct..

[8] Han L.-H., Li W., Bjorhovde R. (2014). Developments and advanced applications of concrete-filled steel tubular (CFST) structures: Members. J. Constr. Steel Res..

[9] Al-Mekhlafi G.M., Al-Osta M.A., Sharif A.M. (2020). Behavior of eccentrically loaded concrete-filled stainless steel tubular stub columns confined by CFRP composites. Eng. Struct..

[10] Tao Z., Han L.H., Wang L.L. (2007). Compressive and flexural behaviour of CFRP-repaired concrete-filled steel tubes after exposure to fire. J. Constr. Steel Res..

[11] Liu L., Lu Y.Y. (2010). Axial bearing capacity of short FRP confined concrete-filled steel tubular column. J. Wuhan Univ. Technol. Mater.

[12] Guan Z.W., Al-Husainy A.S., Wang Q.Y., Jones S.W., Su C., Liu L.Q. (2020). Numerical Modeling of Recycled and Normal Ag-gregate CFRP-Strengthened Concrete-Filled Steel Columns Subjected to Lateral Impact. J. Compos. Constr..

[13] Wang Y.H., Wang Y.Y., Hou C., Deng R., Lan Y.S., Luo W., Li P. (2020). Torsional capacity of concrete-filled steel tube columns circumferentially confined by CFRP. J. Constr. Steel Res..

[14] Wang Q.-L., Li J., Shao Y.-B., Zhao W.-J. (2015). Flexural Performances of Square Concrete Filled CFRP-Steel Tubes (S-CF-CFRP-ST). Adv. Struct. Eng..

[15] Han L.H. (2016). Concrete Filled Steel Tubular Structures—Theory and Practice.

[16] Wang Y.-H., Zhou X.-H., Deng R., Lan Y.-S., Luo W., Li P., Yang Q.-S., Ke K. (2021). Coupled ultimate capacity of CFRP confined concrete-filled steel tube columns under compression-bending-torsion load. Structures.

[17] Elremaily A., Azizinamini A. (2002). Behavior and strength of circular concrete-filled tube columns. J. Constr. Steel Res..

